# SNP/RD Typing of *Mycobacterium tuberculosis* Beijing Strains Reveals Local and Worldwide Disseminated Clonal Complexes

**DOI:** 10.1371/journal.pone.0028365

**Published:** 2011-12-05

**Authors:** Anita C. Schürch, Kristin Kremer, Amber C. A. Hendriks, Benthe Freyee, Christopher R. E. McEvoy, Reinout van Crevel, Martin J. Boeree, Paul van Helden, Robin M. Warren, Roland J. Siezen, Dick van Soolingen

**Affiliations:** 1 Tuberculosis Reference Laboratory, National Institute for Public Health and the Environment (RIVM), Centre for Infectious Disease Control, (CIb/LIS, pb 22), Bilthoven, The Netherlands; 2 Radboud University Nijmegen Medical Centre/NCMLS, Centre for Molecular and Biomolecular Informatics, Nijmegen, The Netherlands; 3 Department of Science and Technology, National Research Foundation Centre of Excellence in Biomedical Tuberculosis Research, Medical Research Council Centre for Molecular and Cellular Biology, Stellenbosch University, Tygerberg, Cape Town, South Africa; 4 Department of Microbiology and Immunology, University of Melbourne, Victoria, Australia; 5 Department of Medicine, Radboud University Nijmegen Medical Centre, Nijmegen, The Netherlands; 6 Department of Pulmonary Diseases, Radboud University Nijmegen Medical Centre/University Lung Centre Dekkerswald, Nijmegen, The Netherlands; Swiss Tropical and Public Health Institute, Switzerland

## Abstract

The Beijing strain is one of the most successful genotypes of *Mycobacterium tuberculosis* worldwide and appears to be highly homogenous according to existing genotyping methods. To type Beijing strains reliably we developed a robust typing scheme using single nucleotide polymorphisms (SNPs) and regions of difference (RDs) derived from whole-genome sequencing data of eight Beijing strains. SNP/RD typing of 259 *M. tuberculosis* isolates originating from 45 countries worldwide discriminated 27 clonal complexes within the Beijing genotype family. A total of 16 Beijing clonal complexes contained more than one isolate of known origin, of which two clonal complexes were strongly associated with South African origin. The remaining 14 clonal complexes encompassed isolates from different countries. Even highly resolved clonal complexes comprised isolates from distinct geographical sites. Our results suggest that Beijing strains spread globally on multiple occasions and that the tuberculosis epidemic caused by the Beijing genotype is at least partially driven by modern migration patterns. The SNPs and RDs presented in this study will facilitate future molecular epidemiological and phylogenetic studies on Beijing strains.

## Introduction

The tubercle bacillus is one of the most important human bacterial pathogens, with an estimated 9.4 million incident cases of tuberculosis globally in 2009 [1]. Using molecular genotyping methods numerous genotypes of *Mycobacterium tuberculosis* have been identified [2], [3]. The Beijing genotype is one of the most studied genotypes, and causes approximately 50% of the tuberculosis cases in Asia [4]. Beijing strains are also a major driving force behind the multidrug-resistant tuberculosis epidemic in Eastern Europe and South Africa [5], [6] and “Typical Beijing” strains may be able to circumvent the BCG vaccine-induced immunity [7], [8].

The *M. tuberculosis* Beijing genotype is easily identified by a highly characteristic spoligotype pattern, resulting from the deletion of the RD207 [9], [10], [11]. Strains of the Beijing genotype were previously grouped into two lineages; “Typical” and “Atypical” [11] according to the presence or absence of an IS*6110* insertion in the NTF region [12]. Strains of the “Typical Beijing” lineage can be defined by the presence of 51 SNPs [13] and form a monophyletic group, whereas strains that were formerly indicated as “Atypical Beijing” were shown to be genetically diverse and paraphyletic and do not form a separate lineage [13]. We address both groups of Beijing strains as “Beijing genotype”. Strains of the dominant “Typical Beijing” lineage are isolated from about 80% of Beijing-infected cases [13] however, the ratio of “Typical” versus other Beijing strains differs significantly by region [7], [14], [15]. The lack of genetic diversity, especially among the “Typical Beijing” strains, points to a recent, clonal expansion of this lineage [13].

Whole genome sequencing and subsequent SNP typing is a method which could be used to analyze the population structure of clonal bacterial pathogens that lack genetic diversity [16]. Due to the recent progress in sequencing technologies [17], [18] and high-throughput SNP typing approaches [19], an increasing number of SNP typing systems have been developed from whole-genome sequencing data of bacterial pathogens [20], [21], [22], [23], [24], [25]. *M. tuberculosis* is a highly clonal microorganism, and no recent horizontal gene transfer or recombination events between different strains have been identified so far [26], [27], [28]. Given the recent ancestry of the *M. tuberculosis* complex (MTBC) and low selective pressure on particular loci, SNPs and RDs (if independent from IS*6110*-directed recombination) represent unique events at unidirectional time points in the genealogy of a *M. tuberculosis* strain. Synonymous SNPs and RDs are representative of ancestral states of strains and can act as molecular markers for clonal complexes. Here, we applied SNPs and newly identified RDs to characterize the population structure of the Beijing genotype strains.

## Results

### Development of SNP/RD typing assay for Beijing strains

To validate identified genome-wide variations and to identify informative and robust markers that could support studies on the phylogeny of the Beijing genotype of *M. tuberculosis* through a redundancy analysis, we used SNPs identified from whole genome sequence comparison of six Beijing strains originating from China, Vietnam, and South Africa, as described in detail in a recent paper [13]. The 275 SNPs selected represented 14.6% of the total 1889 SNPs identified and 21.3% (of 1294 SNPs) of the Beijing-specific- or polymorphic SNPs.

Typing of 178 strains with 275 SNPs in a high-throughput mass-spectrometry typing assay resulted in a total of 48,950 SNP positions being analyzed. A small subset of 289 SNP positions (0.59%) was undetermined and 51 positions (0.1%) had an ambiguous result (both indicated as “?” in Table S1). From the SNP matrix a phylogenetic tree was inferred using maximum likelihood (Figure S1-A). The same phylogeny inferred with maximum parsimony exhibited a consistency index of 0.93, which can be caused by either homoplasious or erroneous SNPs (i.e. due to technical failures). Non-clonally distributed SNPs in the tree included a SNP in *katG* which is known to be involved in antibiotic resistance [29], six other non-synonymous SNPs (in genes *pta*, *rpsL*, *lipU*, *lppF*, *eis* and *serA1*) and a synonymous SNP (in gene *cysA3).* Information on drug resistance frequencies between Typical and other Beijing strains was previously reported in [13].

Two duplicate isolates, that were used to confirm the consistency of the SNP typing (isolates NLA000200230 and NLA009701940) clustered at the same node. Moreover, some genome-sequenced strains were also typed with the SNP typing assay. As expected, the *in silico* determined SNP type of strain CHIN- (that corresponds to strain NLA000700872) was consistent with the high-throughput mass-spectrometry-typed isolate NLA000700872, as was CHIN+ consistent with NL000700873 and SA+ with SAWC5527 [13]. Strain V+ clustered slightly different from its counterpart NLA000800162. After exclusion of the eight non-clonally distributed SNPs from the SNP matrix, the strains V+ and NLA000800162 clustered at the same node of the maximum-likelihood tree (Figure S1-B) which lead us to believe that these SNPs were at least partially the results of errors of the SNP detection assay. However, genes involved in drug resistance are known targets of strong selective pressure which could lead to independent occurrence of a polymorphism on several branches of a tree [30].

**Figure 1:**
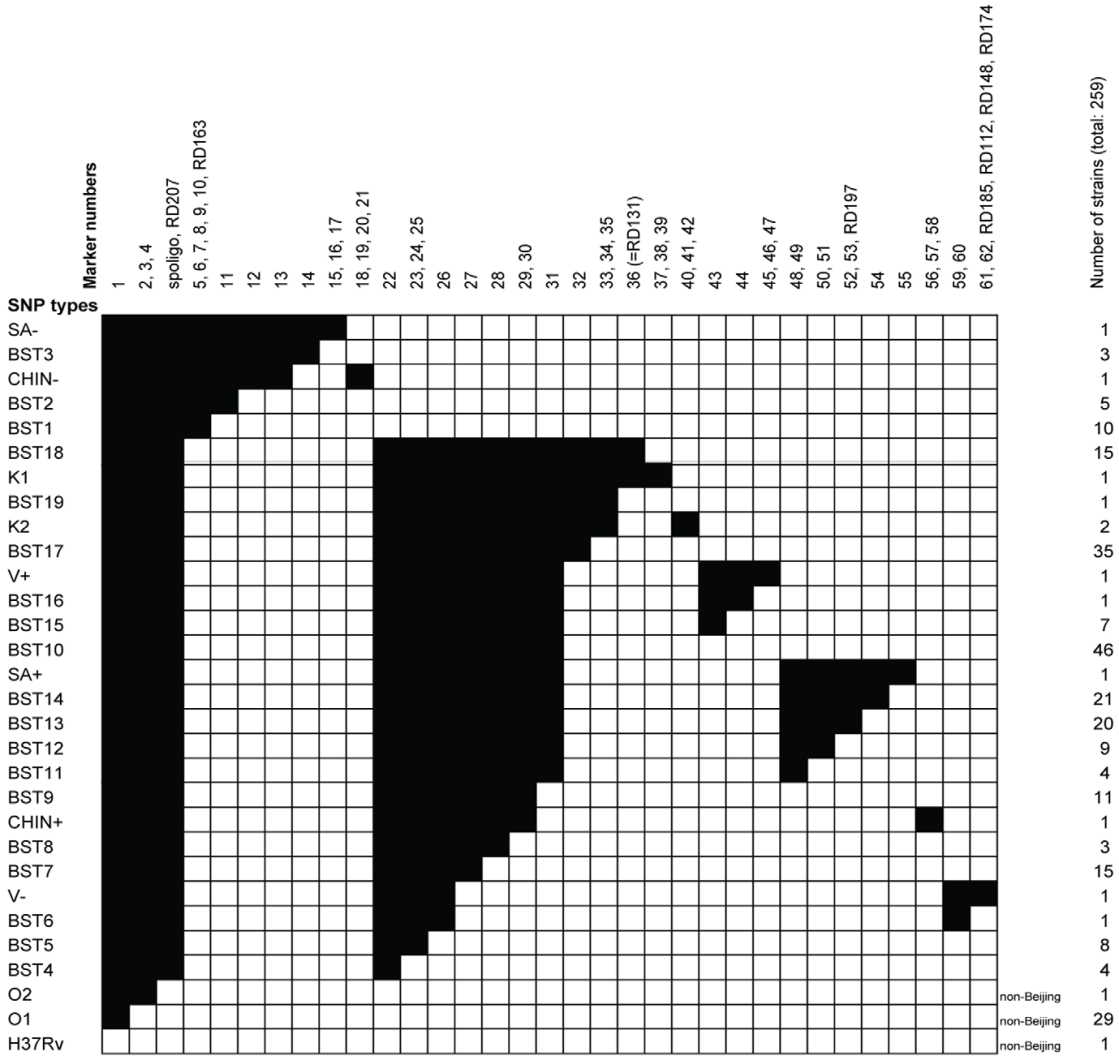
SNP/RD typing results matrix. Matrix of single nucleotide polymorphisms (SNPs)/region of difference (RD) typing scheme results for different clonal complexes of *M. tuberculosis* complex (outgroups H37Rv, O1, O2 and Beijing-SNP types BST1 to BST19 and SA−, CHIN−, V−, SA+, V+, CHIN+, K1 and K2). For the positions and polymorphic sites of the SNPs see Table S2, for details on RDs see Table 2. Black: SNP is present or RD is absent. SNPs and RDs that confer the same information are summarized in one column.

To develop an assay that types Beijing strains in a reliable and efficient way we reduced the number of the 275 SNPs initially selected to type Beijing strains to 51 in a redundancy analysis as described in Material and Methods. During the course of our study, Niemann and colleagues [31] reported the genome sequence analysis of two Beijing strains from Uzbekistan. In order to reduce branch collapse [32], [33] we selected ten SNPs identified in the Beijing strains from Uzbekistan (K1 and K2) [31]. The genomes of these two strains differed by 130 SNPs and one deletion [31] that we named RD131, according to the naming scheme of Tsolaki and colleagues. Table S2 lists the 61 SNPs in detail and Figure 1 shows the SNP matrix.

### Application of SNP/RD typing assay for Beijing strains

These 61 SNPs were assayed on 259 MTBC strains with the high-throughput mass-spectroscopy method (Table S3 and Figure S1-C). The clonal complexes were defined by collecting strains with the same patterns of presence and absence of the markers as indicated in Figure 1. Non-Beijing SNP types were called H37Rv, O1 (outgroup 1) and O2 (outgroup 2), where O2 contains the isolate with the complete spoligotype (all 43 direct variable repeats) and O1 consisted of the sets of MTBC- and non-Beijing strains described in Material and Methods. The inclusion of information on the absence/presence of RD131 (described below in more detail) divided one clonal complex into two distinct complexes (Beijing SNP/RD types (BST) BST18 and BST19). RD131 was, therefore, included in the SNP/RD matrix (Figure 1, number 36), allowing 27 Beijing clonal complexes to be distinguished. These clonal complexes are represented by types BST1 to BST19, and the types with the name of the respective genome-sequenced isolate (Figure 1). The presence of marker 29 and 30 indicate Typical Beijing strains. The SNP/RD assay showed an overall discriminatory power (D) of 0.9.

### Comparison of SNP/RD typing and Beijing lineage designation

For 58 isolates from South Africa, SNP/RD typing results were compared to lineage designation (see Material and Methods). The South African lineage designation [15] had a better discriminatory power for isolates that clustered at SNP/RD types BST7 and BST10, which were differentiated into three and two lineages. The SNP/RD typing system achieved a higher discriminatory power for strains in lineage 6 and 7, which were split up in three SNP/RD types each (Table 1). Overall, the SNP/RD assay had a better discriminatory power compared to the lineage designation on this specific set of isolates (*D = *0.78 versus *D = *0.54).

**Table 1:** Comparison of clustering of 58 *M. tuberculosis* isolates.

*clonal complex*	*SA−*	*BST7*	*BST10*	*BST17*	*BST9*	*BST14*	*BST13*	*SA+*
Lineage								
**1**	1							
**2**		2						
**3**		1						
**4**		1						
**5**			4					
**6**			3	7	1			
**7**						20	17	1

Compared were isolates of the Beijing genotype from South Africa, clustered by the lineage designation of Hanekom et al. (Hanekom et al., 2007) to the clonal complex designation resulting from SNP/RD typing in this study. The numbers in the table represent the number of strains in the respective clonal complex/lineage.

### Comparison of SNP/RD typing and RFLP typing of K1 and K2

In general the isolates represented the diversity of RFLP patterns within the Beijing genotype. Beijing strains K1 and K2 however exhibited an identical IS*6110* RFLP pattern [31] and were isolated in the same geographical region. With the application of RD131 it was possible to identify 15 strains that share a more recent ancestor with K1 than K2, despite the identical RFLP pattern of K1 and K2 (for the IS*6110* RFLP pattern see [31]).

### Identification of RDs within Beijing sublineages

The distribution of RDs known to be polymorphic among Beijing genotype strains (RD105, RD142, RD149, RD150, RD152, RD207 and RD181) in the genome sequenced strains was previously described [13]. In this study we investigated the occurrence of newly identified RDs among Beijing genotype strains (Table 2). *In silico* analysis identified DNA fragments that were absent in one or more of the genome-sequenced strains when compared to the reference strain H37Rv which had not been previously described [9], [10], [34], [35], [36], [37]. These RDs were assayed on a selection of the 259 Beijing strains for absence or presence of RDs with PCR and gel electrophoresis. The strains were selected based on their DNA availability. The strain selection and the results for each of the assayed RDs are shown in the Figures S2, S3, S4, S5, S6 and S7. In summary, the patterns of presence and absence of seven RDs was concordant with the grouping on basis of SNP patterns for all strains assayed. The presence/absence pattern of one RD, RD131 (see above) differentiated one of the clonal complexes defined by SNP typing (BST18 and BST19). The chromosomal position and occurrences of all RDs are summarized in Table 2.

**Table 2:** Regions of difference (RD) identified in *M. tuberculosis* Beijing strains.

Position in H37Rv	Reference	Genes (partially) deleted	RD name	Co-occurrence with marker (Figure 1)	Corresponding Figure
859243 – 859496	[31]	Rv0766c	RD131	36	S2
3120521– 3127920	[9].	Rv2814c – Rv2820c	RD207	spoligo	S3
2949906–2955132	this study	Rv2623 – Rv2627c	RD197	52, 53	S4
2626969–2633061	this study	Rv2394c – Rv2350c	RD185	61, 62	S5
358030–363748	this study	Rv0294–Rv0299	RD112	61, 62	S6
1715870–1733378	this study	Rv1522c–Rv1531	RD148	61,62	S6
2238647–2242137	this study	Rv1995–Rc1997	RD174	61, 62	S6
2128379–2129584	this study	Rv1878	RD163	5, 6, 7, 8, 9, 10	S7

### Phylogeography of the Beijing genotype

To assign a strain to a particular geographic, area the country of origin was indicated for strains isolated in countries other than the Netherlands. For isolates from the Dutch database the patient's country of origin which often coincides with the country of birth of the patients was used.

**Figure 2:**
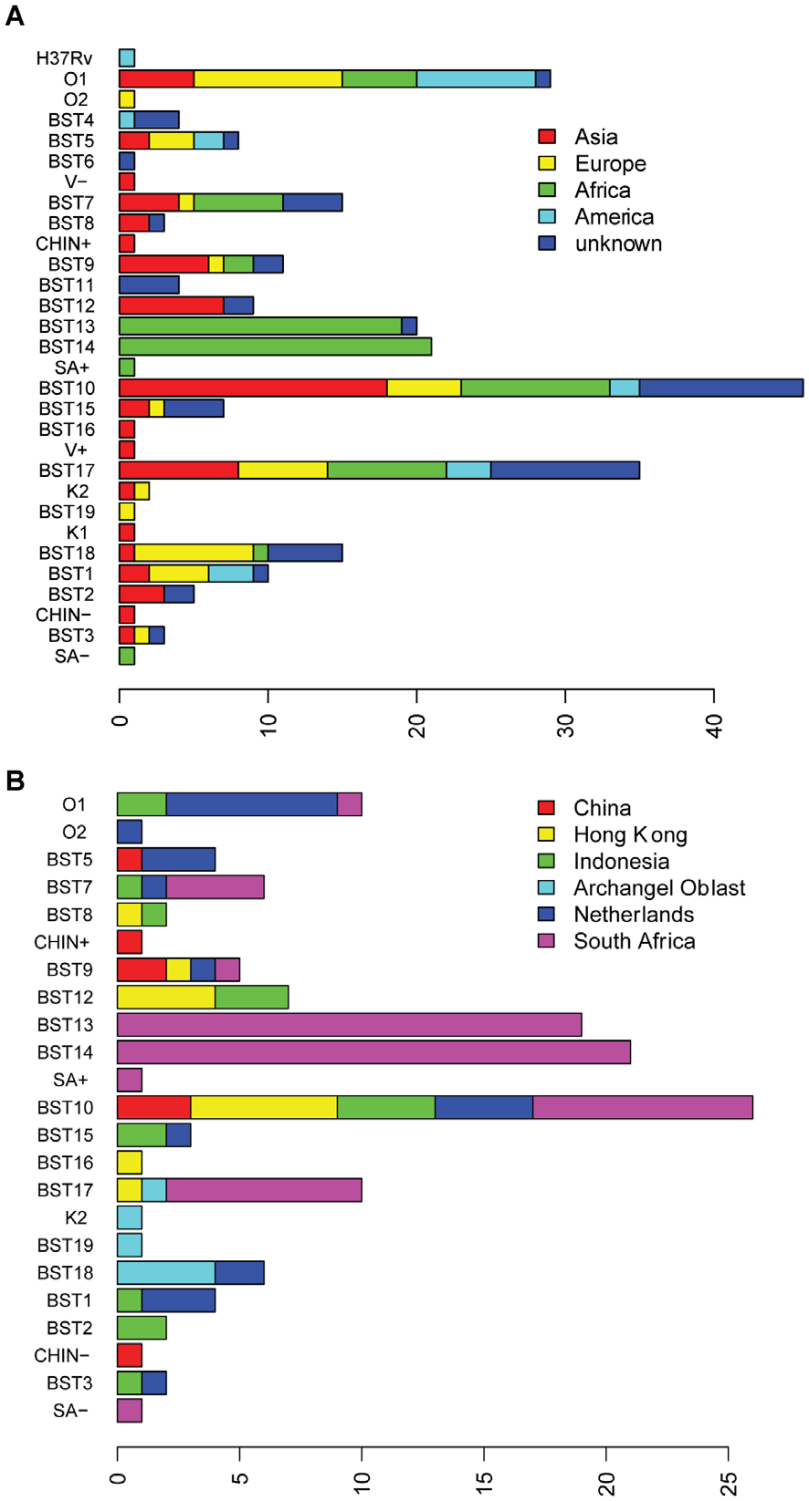
Origins of clonal complexes. A. Continent of origin of the *M. tuberculosis* isolates belonging to clonal complex H37Rv, O1, O2 and BST1 to BST19 and SA−, CHIN−, V−, SA+, V+, CHIN+, K1 and K2. “America” includes isolates from the South- and North American continent. B. Distribution of country of origin within clonal complexes of the *M. tuberculosis* isolates. Here, only isolates belonging to countries or regions with more than five isolates assayed in this study were considered.

The typed *M. tuberculosis* strains originated from across the globe: 68 strains were isolated in Asia, 43 strains in Europe (including three strains from countries that are also on the Asian continent, Azerbaijan and Russia), 74 from Africa, (of which 65 from South Africa), 20 from North- or South America or the Caribbean and 54 with an unknown origin (Figure 2A and Table S4).

All but two clonal complexes that comprised more than one isolate were associated with diverse geographic origins (Figure 2). On the other hand, all isolates from the Archangel Oblast region that were part of this study (Figure 2B) were from clonal complexes that have SNP 32 present, showing that a wide distribution of the clonal complexes is not necessarily coupled to a wide variety locally. However, even a clonal complex that is highly resolved with 13 SNPs present, the K2 clonal complex, comprised two isolates from different origins. This clonal complex consists of isolate K2 (from Uzbekistan) and an isolate from the Archangel Oblast region. The distance between these two locations is more than 2000 km.

In contrast, two clonal complexes (BST13 and BST14) contain only samples from South Africa. These isolates belong to a group of Beijing strains that were associated with an increased transmissibility and ability to cause disease in South Africa (lineage 7, [15]). The more basal clonal complex BST12, which constitutes a clonal complex ancestral to the BST13/BST14 lineage, comprised three isolates from Indonesia and four from Hong Kong, along with two Beijing strains from an unknown origin. These isolates share a common ancestor with the South African strains from BST13 and BST14. Whether this ancestor originated in one of the three countries or a fourth unknown country remains unresolved.

## Materials and Methods

A scheme of the workflow can be found as Figure S8.

### Selection of SNPs

Whole genome sequences from eight Beijing genotype *M. tuberculosis* isolates representing “Typical” (+) and other (−) Beijing strains [11], [31] were analyzed to identify SNPs and regions of difference (RDs). These Beijing strains originated from four different countries; China (CHIN+ and CHIN−), South Africa (SA+ and SA−), Vietnam (V+ and V−) and Uzbekistan (K1 and K2) [13], [31]. H37Rv was used as the reference genome for SNP calling.

From the 1889 SNPs that were identified in a previous study [13], 275 SNPs were selected for a SNP typing assay. The selected SNPs were annotated to be in coding regions, with a preference for characterized genes as opposed to genes that are annotated as “hypothetical gene”. The selection contained only SNPs that did not have any other SNP closer than 500 bp, to avoid hypervariable regions and mutational hotspots. From this selection, 34 SNPs were located in 3R genes (genes involved in DNA repair, recombination and replication [38], [39], [40], [41]). The SNP selection by Mestre et al. [41] based on 3R genes is the current best selection for SNP typing of Beijing strains but has only low discriminatory power within Typical Beijing strains. Of all 275 SNPs, seven SNPs were H37Rv-specific (only present in H37Rv), 10 SNPs were Beijing-specific (with *Mycobacterium bovis* as outgroup), and 258 SNPs were polymorphic among the six Beijing genomes (CHIN+, CHIN−, SA+, SA−, V+, V−). A total number of 168 SNPs were annotated as non-synonymous and 107 SNPs were synonymous.

### Strain selection

One-hundred and seventy-two Beijing genotype strains were selected to cover the diversity of IS*6110* RFLP patterns within the Beijing genotype [11] and comprised strains previously characterized with other markers [42], [43], [44]. The selected strains were representative of 45 countries on five continents: they were isolated in that country or isolated from a patient born in the respective country. H37Rv and five strains from other genotypes were included. Each strain was genotyped using the selected 275 SNPs.

One-hundred and fifty-nine of these strains were subsequently tested in a second assay of 61 informative SNPs (Table S2) and these strains were complemented with five Beijing strains from the same database that met the same inclusion criteria (representing different countries and the diversity of RFLP patterns) as described above. In addition, the 61 SNPs were assayed on 11 additional *M. tuberculosis* Beijing strains from Indonesia (in addition to six strains from Indonesia that were already present in the collection, total Indonesian strains n = 17) and 55 additional Beijing strains from South Africa (in addition to ten South African strains that were already present in the Dutch database, total South African strains n = 65). These 55 strains represented the 7 Beijing lineages described by Hanekom et al. [15] and the abundance of the different Beijing strains in South Africa. To determine the specificity of the markers for the Beijing genotype, an additional two sets of strains consisting of 13 strains of other species within the MTBC; including *Mycobacterium africanum* (n = 2), *Mycobacterium bovis* (n = 6), *M. bovis* BCG (n = 2), *Mycobacterium canettii* (n = 1) and *Mycobacterium microti* (n = 2) and 14 *M. tuberculosis* genotypes other than Beijing were included. One of these strains exhibited a complete spoligotype; such a strain is regarded to be closely related to Beijing strains as shown by large sequence polymorphisms [45]. Furthermore SNP data of two genome-sequenced strains K1 and K2 were included [31]. In total, 259 MTBC strains were assayed using 61 SNPs.

### High-throughput SNP typing and clustering

Bacterial isolates were typed on the Sequenom genotyping platform (Sequenom GmbH, Hamburg, Germany) with iPLEX Gold biochemistry. To this end, primer extension reactions were carried out using oligonucleotide primers designed with the AssayDesigner 3.1 software. Single base extension of a primer that annealed directly adjacent to the SNP was measured on a compact MALDI-TOF mass spectrometer, following automated protocols in a 384-well format [46]. The SNP positions of the genome-sequenced strains (CHIN+, CHIN−, SA+, SA−, V+, V−, K1 and K2) were extracted from the literature [13], [31].

The results of the high-throughput SNP typing for the 275 SNPs or the 61 SNPs were concatenated for each isolate and treated as alignments. A phylogenetic tree was inferred with maximum-likelihood using Phyml version 2.4.4 [47]. The trees were visualized as cladograms in Figures S1-A, S1-B and S1-C with Dendroscope [48]. Subsequently, for each internal and external branch of the phylogenetic tree that was established from 275 SNPs, SNPs were identified that represented the respective branch by their presence or absence in the isolates. To determine the consistency index, a tree was established with maximum-parsimony in MEGA 5 [49]. Non-clonally distributed SNPs in the tree were determined with mixed method discrete character parsimony carrying out Camin-Sokal parsimony as implemented in the Phylip suite (Phylip 3.69).

### Redundancy analysis

SNP results of the assay with 275 SNPs were classified as reliable if no ambiguous bases were detected by the assay and could not be determined less than twice in the different strains (after isolates with more than ten empty SNP positions were excluded). Moreover we did not select the eight SNPs that were potentially the result of convergent evolution or SNP detection errors. Synonymous SNPs were chosen as these are generally assumed to be selectively neutral. We included one coding SNP that was not part of the first assay, but that was representative for a group of four SNPs that marked a short internal branch in the phylogeny of the six genome-sequenced Beijing strains [13]. This SNP causes a conservative amino acid change (valine-to-alanine substitution) in a probable acid-maltase protein.

Of the SNPs that were either specific for K1 or K2 [10] or present in both strains, but not reported in CHIN+, SA+, V+, SA−, CHIN− or V−, ten synonymous SNPs were randomly selected (three specific for strain K1, three specific for strain K2 and four SNPs specific for both K1 and K2).

The nucleotides at the 61 positions in the chromosome of each isolate were arranged in a SNP matrix and positions with the same and therefore redundant information content were merged (see Figure 1). RD131 was treated as one mutation event, and added to the concatenated 61 SNPs.

### Identification and verification of RDs

A BLAST search (BLAST 2.2.19, [50] of all genes of H37Rv with a sliding-window of 200 bp against the raw read collection of six 454/Roche-sequenced Beijing genomes (CHIN−, CHIN+, SA−, SA+, V+, V−, [13] with read lengths of 250 bp on average was performed. Hits with query coverage of less than 0.8 were used to identify potential RDs.

To confirm the RDs identified *in silico* and to assay their presence or absence in selected Beijing isolates, PCR primers were designed adjacent to the putative deletion sites with Primer3plus (http://www.bioinformatics.nl/cgi-bin/primer3plus/primer3plus.cgi) with standard parameters. For RDs larger than 2000 bp, two sets of primers were designed (primer sequences are indicated in Table S5). PCR reactions were performed with either PuReTaq Ready-To-Go™ PCR beads (GE Healtcare LifeScience, Little Chalfont, UK) or HotStarTaq Master Mix (Qiagen, Hilden, Germany) as mono- or multiplex reactions. The sizes of the PCR products were estimated on a 1% agarose gel to specify the presence or absence of the RD. Selected products were sequenced on an ABI 3730x sequencer (Applied Biosystems, Foster City, CA, USA) following standard protocols.

### Discriminatory power

The discriminatory power (*D*) is the average probability that a typing system will assign a different type to two unrelated strains and was calculated according to the method described by Hunter and Gaston [51], [52]. To compare the discriminatory power of the SNP/RD typing system relative to other typing systems, we compared another lineage designation (Lineage 1 to 7, established by Hanekom and colleagues [15]) of 58 isolates from South Africa to our classification in clonal complexes. These 58 isolates comprised three strains from the Dutch database from lineage 7. The lineage designation was based on IS*6110* insertion sites as well as on synonymous SNPs, RDs, and SNPs in mismatch repair genes [15].

## Discussion

We attempted to define a set of evolutionary stable, non-homoplasious genomic changes in the Beijing genotype identified in eight genome-sequenced strains that could serve as phylogenetic markers, and selected nucleotide changes at neutral or nearly-neutral sites. Furthermore, we included newly identified RDs in the SNP/RD typing scheme for Beijing strains, since deletions that occurred independently from IS*6110*-directed recombination have been successfully used to identify lineages in the MTBC [9], [53], [54], [55], [56], [57], [58]. With the application of the 61 SNPs and one RD to MTBC strains, 30 clonal complexes, of which 27 strictly comprised Beijing genotype strains, were distinguished.

A SNP/RD assay has many advantages over spoligotyping, IS*6110* RFLP and VNTR typing. Synonymous SNPs and RDs are phylogenetically robust, and do not suffer from convergent evolution (at least if deletions were independent from repeat regions [59]) and moreover, they are relatively easy to determine by a PCR-based detection method [54]. In this study, the discriminatory power of RDs is low compared to that of the SNPs: only five clonal complexes could be distinguished by the RDs described (*D* = 0.52). Moreover, RDs did not improve the resolution of the SNP-based assay, with one exception (RD131). However, the SNPs from the lineage leading to K1 and K2 have not been selected by a redundancy analysis but stochastically and thus may not provide the optimal discriminatory power that might have been achieved by a different set of SNPs. It is likely that some SNPs exist that can split up the identified clonal complexes, as does RD131. For these reasons we combined SNPs and RDs in one typing scheme.

A particularly striking result of this study is the almost complete absence of strong geographical associations of all but two clonal complexes. This in contrast to phylogeographical studies of diseases such as the plague [60], lepra [22], buruli ulcer [23], and in contrast to the strong phylogeographic association of major tuberculosis strain lineages reported in several studies [28], [61], [62], [63]. In those studies, the Beijing/W lineage was strongly associated with patients of Asian origin. It has been suggested that this lineage has evolved so as to become adapted to the specific host background and that transmission of disease among patients with the same ethnicity is more likely [61]. In our study, patients from Asian origin form the second largest group (with 68 patient isolates) after patients from African origin (74 isolates). However, our study was not designed to include a representative sample from each region, but to include the genetic diversity among the Beijing RFLP patterns in our database combined with varying countries of origin of the isolates. Thus our study could not estimate the prevalence by region, or give information about the likelihood of transmissibility of clonal complexes. Our study does show that most Beijing clonal complexes can be isolated from patients with diverse genetic backgrounds.

The main difference between studies that find strong phylogeographic associations and our study is the scale of genetic diversity of the strains studied. Our results suggest that several Beijing ancestors spread successfully to different parts of the world and to hosts from diverse ethnicity on multiple occasions. One of the driving forces of the Beijing epidemic might be migration patterns which allow the wide distribution of closely related strains, such as air travel. In contrast, two clonal complexes (BST13 and BST14) were populated exclusively with samples from South Africa and show a strong association to a single country. It is unclear whether these strains are adapted to the South African population or if these clonal complexes did not have the chance to spread to other parts of the world yet, because of limited time or the socioeconomic circumstances of their hosts. Recently, van Helden et al. [64] suggested that Beijing strains might have been introduced to South Africa following the sea trade route from East Asia to Europe that started 400 years ago. Indeed, in the 17th and 18th centuries, Dutch colonists at the Cape of Good Hope largely imported slaves from Indonesia, Madagascar, Mozambique, and India. However, while the conjecture that Beijing strains were transported via the slave or trade route is plausible the study design and sampling do not preclude this conclusion.

The application of SNPs and RDs to non-genome-sequenced strains yields linear phylogenies with a complete absence of secondary branches (branch collapse), a phenomenon inherent to SNP-based phylogenies [32], [33], [65]. As expected in a branch-collapsed typing scheme, clonal complexes that are determined with a maximum number of SNPs present discriminate better than SNP types that are defined by fewer markers. These cannot distinguish between different lineages, as exemplified by comparing the lineage designation to the SNP/RD typing scheme. All samples that were typed in this study were more or less contemporary with respect to TB infections (they were isolated between 1993 and 2009). If fully sequenced, all these isolates would be separated by long branches [32], [33]. In addition, the number of SNPs is not representative for the genetic distance between the clonal complexes, because we selected one to six SNPs to mark a clonal complex, regardless of evolutionary distance.

We postulate that, with the increasing number of SNPs identified from whole genome sequencing efforts, the resolution to determine the population structure will increase, and possibly more clonal complexes could show an association with country of origin. This improved discriminatory power will reach its optimum by genome sequencing of all *M. tuberculosis* isolates in the future. It is already clear however, that almost every Beijing clonal complex has disseminated to different parts of the world on multiple occasions. The SNPs and RDs presented in this study could –partially or jointly – be used in typing assays that aid in future molecular epidemiological studies of the Beijing genotype.

## Supporting Information

Figure S1 **Maximum-likelihood trees from SNP data.** A: Maximum-likelihood tree of 275 concatenated SNPs in 178 *M. tuberculosis* complex strains. B: Maximum-likelihood tree of 267 concatenated SNPs in 178 *M. tuberculosis* complex strains. Eight SNPs that were non-clonally distributed in Figure 1A were excluded for this figure. **C**: Maximum-likelihood tree of 61 concatenated SNPs in 259 strains. Outgroups O1 and O2 are indicated.
(PDF)

Figures S2 **Distribution of RD131 in the phylogenetic tree.** Strains with background colors were assayed for the absence or presence of the RD. No background color: strain not assayed. Red: RD is present (deletion was identified). Yellow: RD is absent (no deletion has occurred). Green: product of other size than the expected product. For corresponding RDs see Table 2.
(PDF)

Figure S3 **Distribution of RD207 in the phylogenetic tree.**
(PDF)

Figure S4 **Distribution of RD197 in the phylogenetic tree.**
(PDF)

Figure S5 **Distribution of RD185 in the phylogenetic tree.**
(PDF)

Figure S6 **Distribution of RD112, RD148 and RD174 in the phylogenetic tree.**
(PDF)

Figure S7 **Distribution of RD163 in the phylogenetic tree.**
(PDF)

Figure S8 **Scheme of workflow applied in this study.**
(PDF)

Table S1 SNP matrix of 275 SNPs in 178 *M. tuberculosis* complex strains. 1: derived from 454 sequencing. 2: derived from high-throughput mass-spectrometry typing.?: no or ambigous base determined. The Supporting Tables contain tab-delimited file that can be copied into spreadsheets.
(TXT)

Table S2 61 Single nucleotide polymorphisms and RD 131 (number 36) used in this study.
(TXT)

Table S3 SNP matrix of 61 SNPs in 259 *M. tuberculosis* complex strains.
(TXT)

Table S4 Country of origin of the isolates used in this study.
(TXT)

Table S5 Primer sequences and PCR conditions used to determine regions of difference (RDs). RD 207 was determined by spoligotyping and is therefore not present.
(TXT)
