# Supplementary material for: SNP/RD Typing of Mycobacterium tuberculosis Beijing Strains Reveals Local and Worldwide Disseminated Clonal Complexes
Source: PLoS One. 2011 Dec 5;6(12):e28365. doi: 10.1371/journal.pone.0028365 (PMC3230589; doi:10.1371/journal.pone.0028365)

Supporting Figure S1 **Maximum-likelihood trees from SNP data.**

A: Maximum-likelihood tree of 267 concatenated SNPs in 178 M. tuberculosis complex strains.

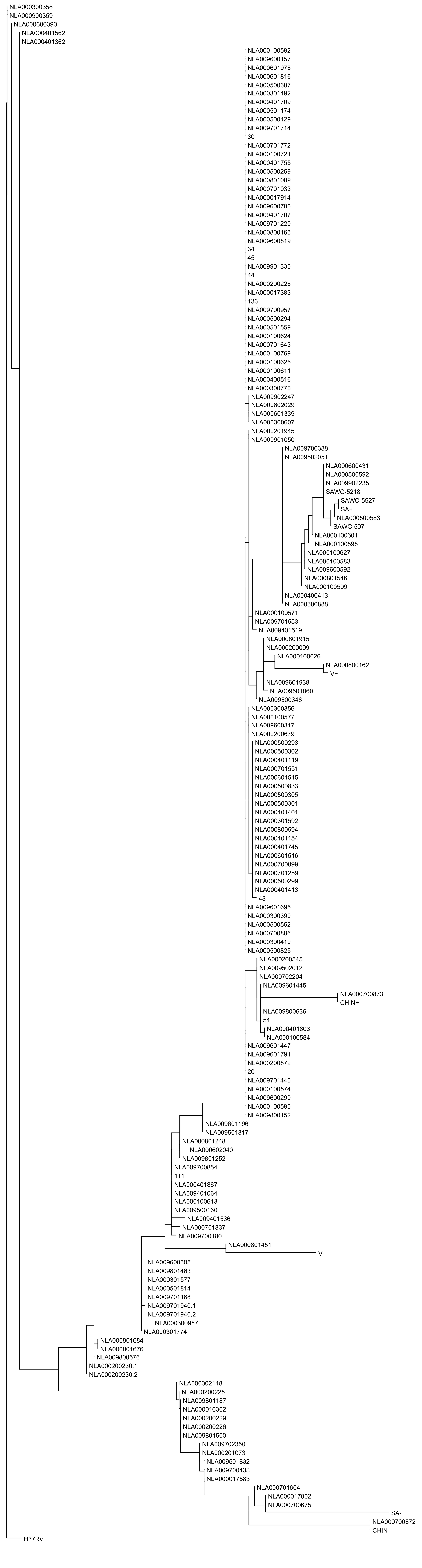

Supporting Figure S1 **Maximum-likelihood trees from SNP data.**

B: Maximum-likelihood tree of 267 concatenated SNPs in 178 M. tuberculosis complex strains.
Eight SNPs that were non-clonally distributed in Figure1A were excluded for this figure.

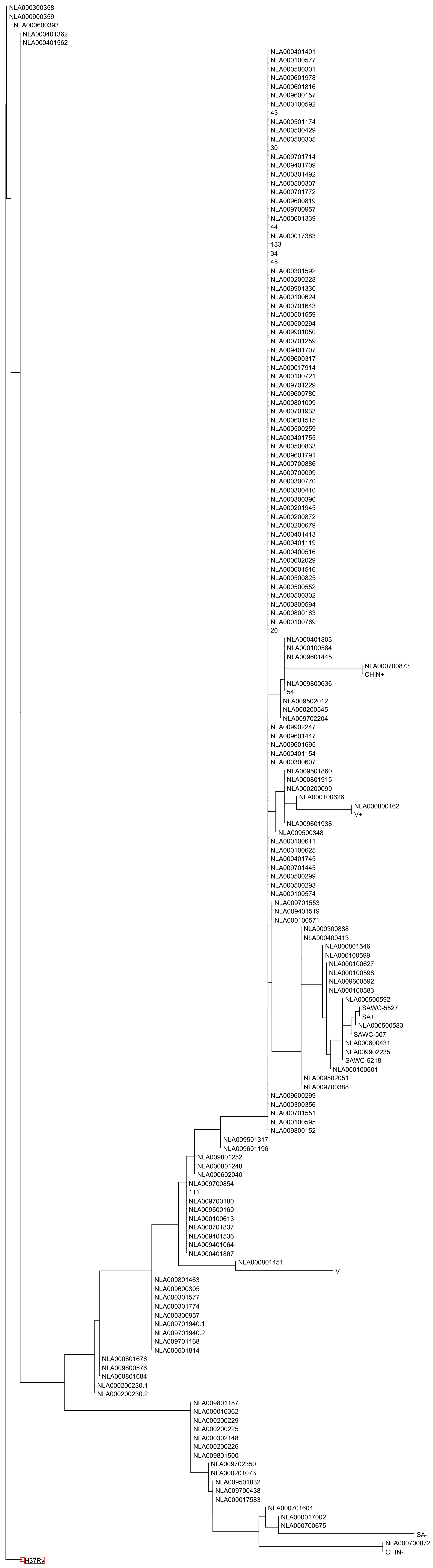

Supporting Figure S1
C: Maximum-likelihood tree of 61 concatenated single-nucleotide polymorphisms (SNPs) in 259 strains.

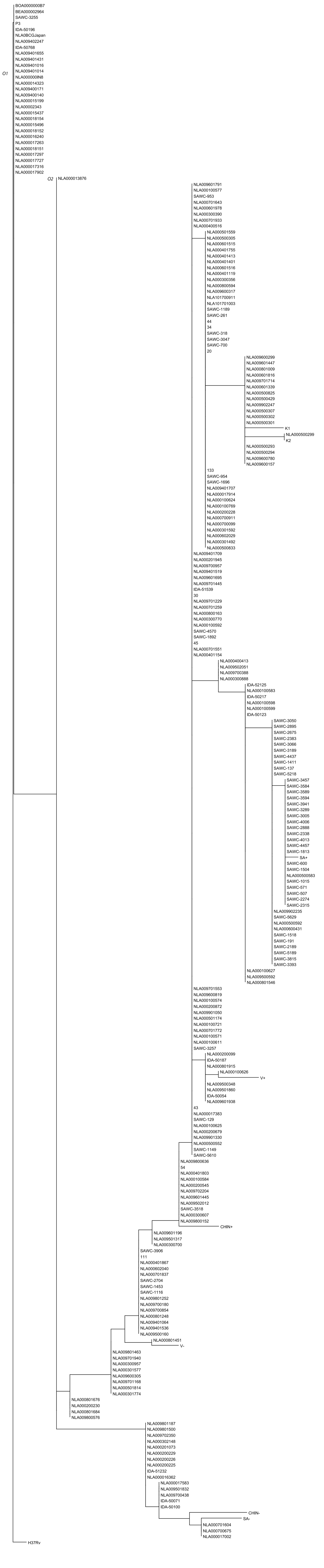

Supplement: Figure S1 — Maximum-likelihood trees from SNP data. A: Maximum-likelihood tree of 275 concatenated SNPs in 178 M. tuberculosis complex strains. B: Maximum-likelihood tree of 267 concatenated SNPs in 178 M. tuberculosis complex strains. Eight SNPs that were non-clonally distributed in Figure 1A were excluded for this figure. C: Maximum-likelihood tree of 61 concatenated SNPs in 259 strains. Outgroups O1 and O2 are indicated. (PDF) [file pone.0028365.s001.pdf]
