# Supplementary material for: SNP/RD Typing of Mycobacterium tuberculosis Beijing Strains Reveals Local and Worldwide Disseminated Clonal Complexes
Source: PLoS One. 2011 Dec 5;6(12):e28365. doi: 10.1371/journal.pone.0028365 (PMC3230589; doi:10.1371/journal.pone.0028365)
Supplement: Figures S2 — Distribution of RD131 in the phylogenetic tree. Strains with background colors were assayed for the absence or presence of the RD. No background color: strain not assayed. Red: RD is present (deletion was identified). Yellow: RD is absent (no deletion has occurred). Green: product of other size than the expected product. For corresponding RDs see Table 2. (PDF) [file pone.0028365.s002.pdf]

Supporting Figure S2 **Distribution of RD131 in phylogenetic tree**
Strains with background colors were assayed for the absence or presence of the RD.
No background color: strain not assayed.
Red: RD is present (deletion was identified).
Yellow: RD is absent (no deletion has occurred).

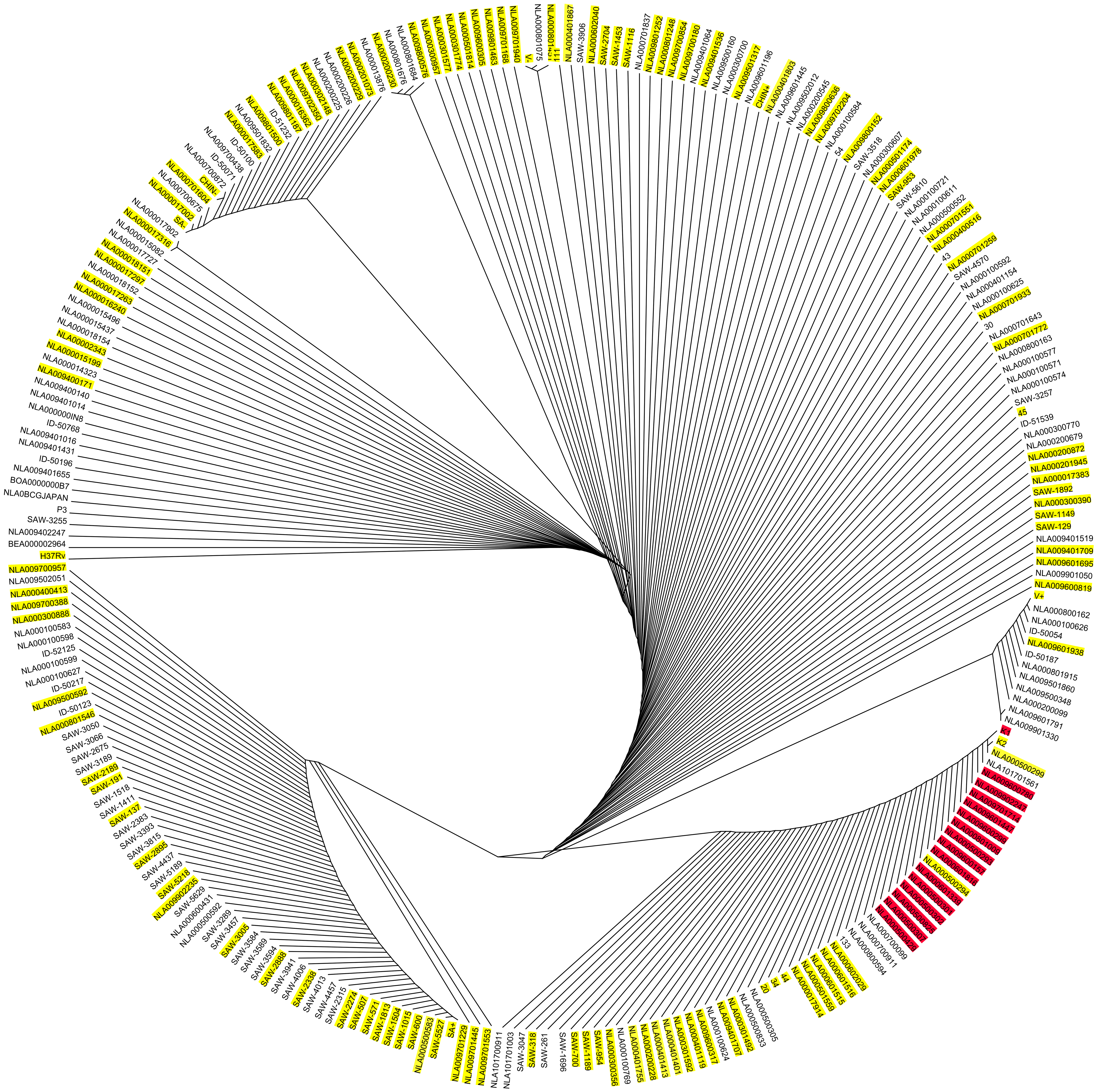

I do not think that it has been shown that mutT genes are really inactivated in Beijings, but there are other options as well.
