# Supplementary material for: SNP/RD Typing of Mycobacterium tuberculosis Beijing Strains Reveals Local and Worldwide Disseminated Clonal Complexes
Source: PLoS One. 2011 Dec 5;6(12):e28365. doi: 10.1371/journal.pone.0028365 (PMC3230589; doi:10.1371/journal.pone.0028365)

Supporting Figure S7, **Distribution of RD163 in the phylogenetic tree**
Strains with background colors were assayed for the absence or presence of the RD.
No background color: strain not assayed.
Red: RD is present (deletion was identified).
Yellow: RD is absent (no deletion has occurred).
Green: product of other size than expected product

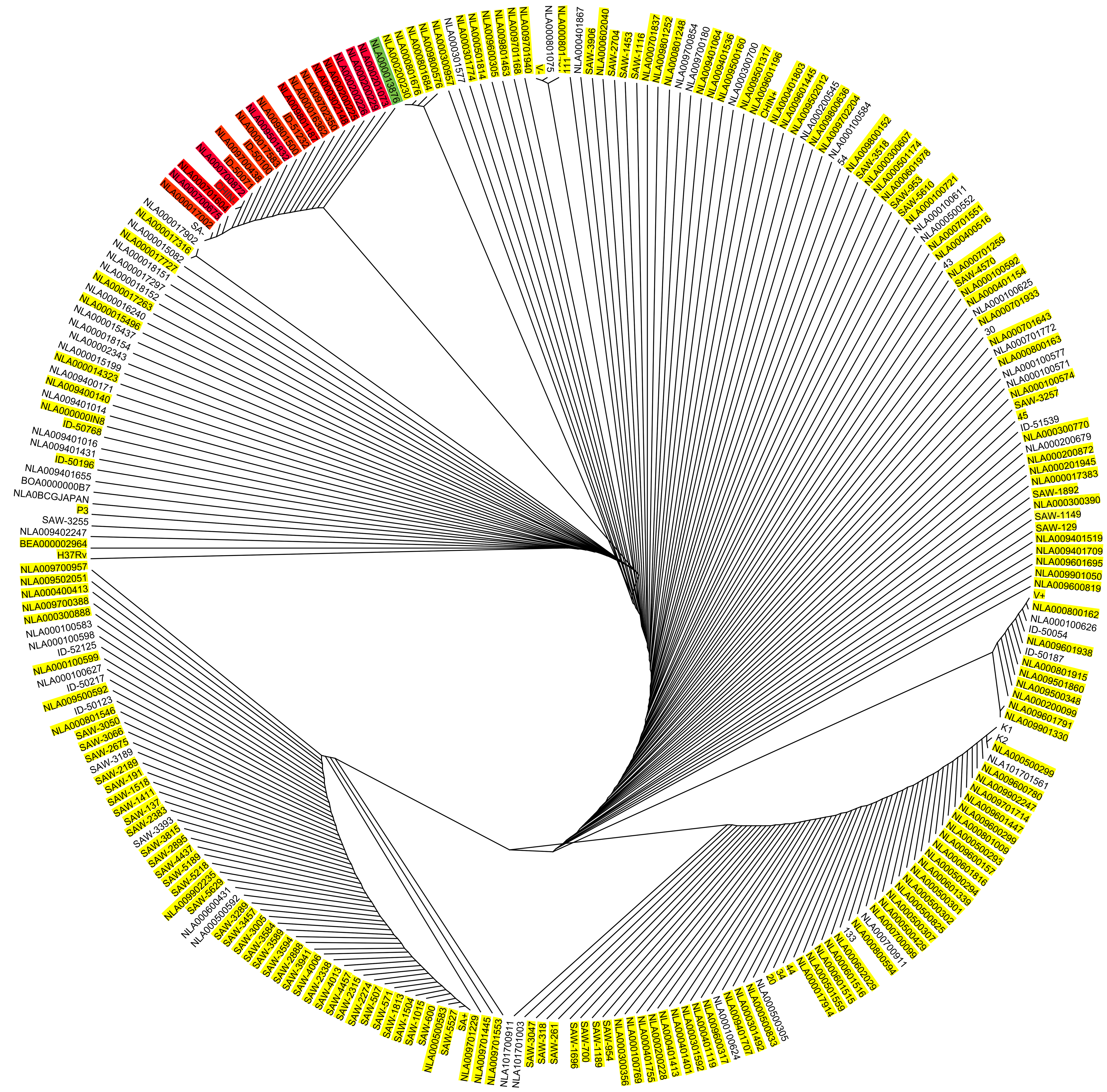

Supplement: Figure S7 — Distribution of RD163 in the phylogenetic tree. (PDF) [file pone.0028365.s007.pdf]
