# Supplementary figures and images for: SNP/RD Typing of Mycobacterium tuberculosis Beijing Strains Reveals Local and Worldwide Disseminated Clonal Complexes
Source: PLoS One. 2011 Dec 5;6(12):e28365. doi: 10.1371/journal.pone.0028365 (PMC3230589; doi:10.1371/journal.pone.0028365)

Figure S8: Scheme of workflow applied in this study

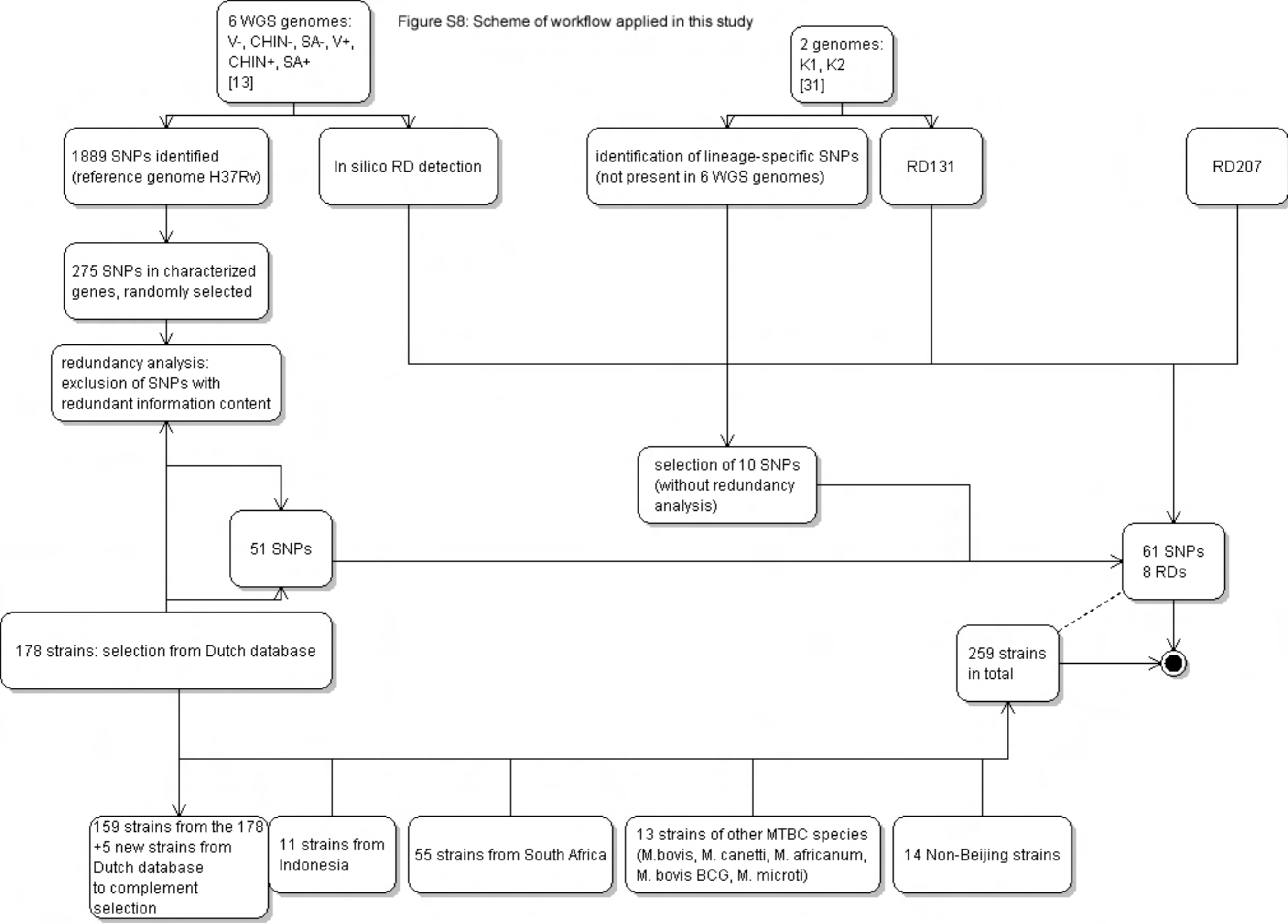

Supplement: Figure S8 — Scheme of workflow applied in this study. (PDF) [file pone.0028365.s008.pdf]
